# When did mammoths go extinct?

**DOI:** 10.1038/s41586-022-05416-3

**Published:** 2022-11-30

**Authors:** Joshua H. Miller, Carl Simpson

**Affiliations:** 1 Department of Geosciences, University of Cincinnati, Cincinnati, OH USA; 2 University of Colorado Museum of Natural History and Department of Geological Sciences, University of Colorado Boulder, Boulder, CO USA

**Keywords:** Palaeoecology, Palaeoecology

arising from Y. Wang et al. *Nature* 10.1038/s41586-021-04016-x (2021)

A unique challenge for environmental DNA (eDNA)-based palaeoecological reconstructions and extinction estimates is that organisms can contribute DNA to sediments long after their death. Recently, Wang et al.^1^ discovered mammoth eDNA in sediments that are between approximately 4.6 and 7 thousand years (kyr) younger than the most recent mammoth fossils in North America and Eurasia, which they interpreted as mammoths surviving on both continents into the Middle Holocene epoch. Here we present an alternative explanation for these offsets: the slow decomposition of mammoth tissues on cold Arctic landscapes is responsible for the release of DNA into sediments for thousands of years after mammoths went extinct. eDNA records are important palaeobiological archives, but the mixing of undatable DNA from long-dead organisms into younger sediments complicates the interpretation of eDNA, particularly from cold and high-latitude systems.

All animal tissues, including faeces, contribute DNA to eDNA records^2^, but the durations across which tissues can contribute genetic information must vary depending on tissue type and local rates of destruction and decomposition. On high-latitude landscapes, soft tissues and skeletal remains of large mammals may persist, unburied, for millennia^3–5^. For example, unburied antlers of caribou (*Rangifer tarandus*) from Svalbard (Norway) and Ellesmere Island (Canada) have been dated^3,4^ to between 1 and 2 cal kyr bp (calibrated kyr before present). Elephant seal (*Mirounga leonina*) remains near the Antarctic coastline^5,6^ can persist for more than 5,000 years. This is in contrast to bones in warmer settings, which persist for only centuries or decades^7,8^. Because bones are particularly resistant to decay, quantifying how their persistence changes across environments enables us to constrain the durations that dead individuals generally contribute to eDNA archives. To do this, we consolidated data on the oldest radiocarbon-dated surface-collected bones from different ecosystems. We included bones that we are reasonably confident persisted without being completely buried (‘never buried’), and bones for which exhumation cannot be confidently excluded (‘potentially never buried’). Pairing bone persistence with mean annual temperatures (MAT) from their sample localities, we find a strong link between the local temperature and the logged duration of bone persistence (Fig. 1, never buried bones: *R*^2^ = 0.94, *P* < 0.01; potentially never buried bones: *R*^2^ = 0.95, *P* < 0.01). Millennial-scale bone persistence is probably ubiquitous in Arctic ecosystems, particularly those with low sedimentation rates. Bone persistence increases with body size^7^, so although the persistence of Arctic mammoth bones is unknown, results based on smaller-bodied organisms in warmer modern temperatures (Fig. 1) are probably underestimates of bone persistence for Pleistocene megafauna living in colder settings. Of note, bones and other biological tissues in cold environments are frozen for much of each year and even weather-worn specimens can produce viable DNA^6^.

**Fig. 1:**
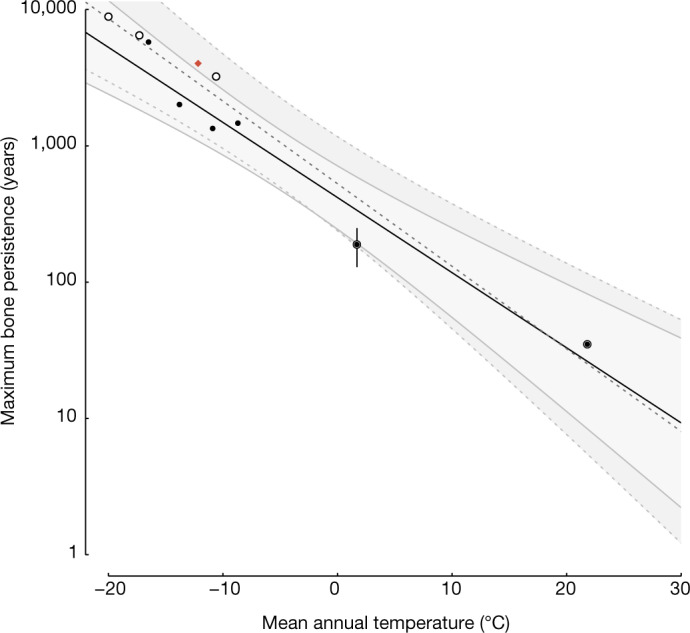
Duration of exposed bone persistence on landscapes as a function of the bone location’s MAT. Persistence estimates (regressions and their 95% confidence intervals) are shown for bones that have probably remained at least partially exposed for their entire post-mortem history (never buried; filled points, solid lines, *R*^2^ = 0.94, *P* < 0.01) and bones that were found exposed, but have more ambiguous post-mortem histories (potentially never buried; open circles, dashed lines, *R*^2^ = 0.95, *P* < 0.01). For locations with more limited sampling, the same bones were used for both regressions (filled points surrounded by open circles). The most recent mammoth bone found exposed on Wrangel Island is shown (red diamond), but is not included in the regressions. Error bars are 2*σ* and generally smaller than the points.

eDNA, like all other sedimentary records, incorporates inputs from many sources and ages^2,9^. Although this temporal mixing is frequently ignored in deference to inputs from living individuals, dead remains also contribute DNA as they decay. The magnitude of temporal mixing in eDNA must, therefore, largely depend on the decay durations of bones and other tissues. Because DNA cannot be directly dated, the degree of temporal mixing cannot be estimated for an individual eDNA sample. However, even diminutive antlers of female caribou can persist on tundra surfaces for more than 3,000 years (Fig. 1). Beyond extended bone persistence, Arctic settings are often characterized by ice-driven (for example, frost-heaving and cryoturbation) and geomorphological processes that release ancient fossils to the surface, thereby expanding the magnitude of temporal mixing within eDNA^10^. Wang et al. themselves reported mammoth DNA from surface samples adjacent to mammoth bones eroding out of nearby sediments^1^. Although they interpret this as contamination today, if this same temporal mixing occurred during the formation of sediment layers from the deeper past, it would go unnoticed.

How much temporal mixing can we expect in eDNA records? Arguably, the best time to evaluate this question is following a species extinction, after which contributions of DNA into sediments shift from a mix of live- and dead-sources to dead-only sources. The timing of extinction is unlikely to coincide with the last occurrence of that species^11^, but the temporal distribution of body fossils or eDNA can be used to estimate extinction timing. Mammoth body fossils found in Northeast Siberia, Northwest and Central Siberia, and northern North America (*n* = 101, 468, and 394, respectively; Supplementary Methods and Supplementary Data 3) are known semi-continuously from around 50 cal kyr bp until their last occurrences. Thus their predicted extinction intervals^12^ (Supplementary Methods) are tightly constrained (Fig. 2). Using eDNA records, we find that extinction intervals are poorly constrained and, for Northwest and Central Siberia, includes the modern day (Fig. 2). More importantly, the mean extinction estimate for Northwest and Central Siberia is 2.7 cal kyr bp. On the basis of the temperature of the most recent mammoth DNA-bearing site (MAT = −13.3 °C), we would expect bone persistence times of between 2.26 and 4.19 kyr (mean and upper 95% confidence intervals for never buried bones) to more than 8.0 kyr (upper 95% CI for potentially never buried bones). Thus, using eDNA time series at face value implies that bones of the last mainland Siberian mammoths might still be persisting on today’s landscapes. Yet, in the face of concerted efforts, the most recent mammoth fossils in this region are no younger than 11 cal kyr bp and are generally entombed in permafrost^10,13^. This differs from Wrangel Island (expected bone persistence between 1.96 kyr and 3.53 kyr (mean and upper 95% confidence interval for never buried bones) to more than 6.66 kyr upper 95% confidence interval for potentially never buried bones), where mammoths persisted until 4 cal kyr bp, Middle Holocene sediments are thin and their bones lie exposed on the ground^14^.

**Fig. 2:**
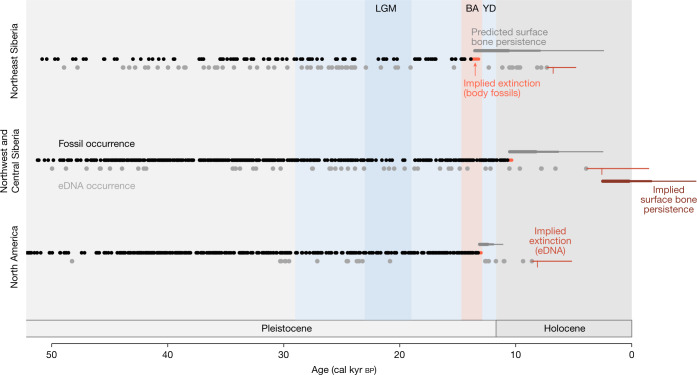
Time series of mammoth body fossils and eDNA records. Body fossils (black points) and eDNA (grey points) are illustrated separately. The 95% confidence intervals for mammoth extinctions are estimated^12^ separately for fossil and eDNA records^1^ in each region (red horizontal lines; vertical line is mean extinction estimate using eDNA records). Predicted persistence of mammoth bones for each region extends from the median of the bone-informed extinction estimate. Thick grey horizontal lines, mean prediction based on never buried bones; medium grey horizontal lines, upper 95% confidence interval based on never buried bones; thin grey horizontal lines, upper 95% confidence interval for potentially never buried bones. LGM, last glacial maximum; BA, Bølling Allerød; YD, Younger Dryas.

One possibility is that millennial-scale gaps between the last mammoth fossils and the youngest eDNA samples highlight the inherent incompleteness of fossil records. This seems to be an unlikely driver, given the near-continuous record of mammoth fossils (Fig. 2) that terminate without a recognized sedimentological shift. eDNA might also be recording individuals immigrating from Holocene mammoth populations on Wrangel Island or the Pribilof Islands. This too seems unlikely, given the wide oceanic crossings that would be required^15^. Instead, we consider the most parsimonious explanation to be that mammoth-bearing Middle Holocene sediments incorporated genetic information from well-preserved remains still lying on landscapes or introduced from exhumed remains of even more ancient individuals. This explanation is corroborated by our finding that the ages of all Siberian sediments containing mammoth DNA are within the expected interval between the last mammoth occurrences and the durations those remains could persist on Siberian landscapes (Fig. 2). Although two North American sediments containing mammoth DNA are younger than expected, exhumation of remains from deeper sediments could explain the genetic occurrence of this extinct species.

Nevertheless, eDNA records of mammoths extend beyond their fossil records. As Wang et al. claim^1^, a possible reason is that mammoths survived on mainland North America and Eurasia into the Middle Holocene. However, the combined evidence indicates that this pattern can be explained by Arctic environmental and taphonomic conditions that increase the persistence of DNA-bearing tissues on landscape surfaces and permit the release of long-dead tissues exhumed from permafrost. The mixing of DNA from long-dead organisms into younger sediments complicates the interpretation of eDNA, but we can start to control for this challenge by assessing the lengths of time across which DNA of extinct species are incorporated into sedimentary records.

## Methods

To evaluate how bone persistence durations change with environment, we aggregated literature records of the ages of bones collected from landscape surfaces. For the purposes of this study, we only included the oldest bone from each region. To diversify the environmental settings included in the dataset, we added three accelerator mass spectrometry radiocarbon-dated bones from Arctic Alaska (two caribou antlers from the Coastal Plain, Arctic National Wildlife Refuge, USA) and temperate North America (one elk (*Cervus elaphus*) femur from Yellowstone National Park, USA; Supplementary Methods and Supplementary Data 1). For a full description of methods used, see Supplementary Information.

## Reporting summary

Further information on research design is available in the Nature Portfolio Reporting Summary linked to this article.

## Online content

Any methods, additional references, Nature Portfolio reporting summaries, source data, extended data, supplementary information, acknowledgements, peer review information; details of author contributions and competing interests; and statements of data and code availability are available at 10.1038/s41586-022-05416-3.

## Supplementary information

Supplementary Information Supplementary Methods and references

Reporting Summary

Supplementary Data 1 Durations that bones persist on landscapes around the world.

Supplementary Data 2 Estimated MATs for eDNA occurrences that are more recent than the last mammoth fossil. eDNA occurrences from Wang et al.^1^. Includes estimated MAT for Wrangel Island.

Supplementary Data 3 Time series of mammoth fossils from northern North America.

## Data Availability

All data generated or analysed during this study are included in the Article and its supplementary information.
