# Supplementary material for: When did mammoths go extinct?
Source: Nature. 2022 Nov 30;612(7938):E1–3. doi: 10.1038/s41586-022-05416-3 (PMC9712083; doi:10.1038/s41586-022-05416-3)
Supplement: Supplementary file 1 — Supplementary Methods and references [file 41586_2022_5416_MOESM1_ESM.pdf]

nature portfolio

https://doi.org/10.1038/s41586-022-05416-3

Supplementary information

When did mammoths go extinct?

In the format provided by the authors and unedited

Guide to Supplementary Information for "When did mammoths go extinct?"

Supplementary Information contains three sections. (1) Supplementary methods. (2) Supplementary References. (3) Supplementary Data 1-3.

Supplementary Data:

Supplementary Data 1. Durations that bones persist on landscapes around the world.

Supplementary Data 2. Estimated mean annual temperatures for eDNA occurrences that are more recent than the last mammoth fossil. eDNA occurrences from Wang et al. 2021[1]. Includes estimated mean annual temperature for Wrangel Island.

Supplementary Data 3. Time series of mammoth fossils from northern North America.

Supplementary Information for:

When did mammoths go extinct?

Joshua H. Miller[1*] and Carl Simpson[2]

[1]University of Cincinnati, Department of Geology, [2]University of Colorado Boulder, Department of Geological Sciences.

* Corresponding author, josh.miller@uc.edu

Contents:

1. Supplementary Methods 2
    a. Evaluating bone persistence around the world 2
    b. Estimating temperatures at bone, fossil, and eDNA localities 3
    c. Modelling mammoth extinction using fossil and eDNA time series 3
2. Supplementary References 4

1. Supplementary Methods

a. Evaluating bone persistence around the world.

To identify the duration bones persist in different environments, we used previously published accounts of radiocarbon dated bones with estimated burial histories. We included all regions from which multiple radiocarbon dates are available and/or there was an attempt to document the maximum ages of bones on the landscape. Signs of burial or partial included root etching, corrosion, maceration, and other physical and chemical modifications to surface bone textures[2]. In some cases, bones were found partially buried (ranging from a small depression in soils to cases where the majority of the bone was under a layer of either organic-rich tundra soils or more mineral-rich sediments). Using available descriptions, we identified bones for which we can be reasonably confident that they were never fully buried. We also include bones with burial histories that are more ambiguous. These included bones that were found fully exposed on modern landscapes, but with more heavily modified bone surfaces. While these bones may not have ever experienced complete burial, surface texture modifications made that determination more difficult to assess. These are the bones for which recent exhumation is most possible. We anticipate that if these were, in fact, exhumed, they would have been just under the surface, having been exposed by activities such as digging by ground squirrels or bears, as opposed to being exhumed from greater depths by frost-heaving or other cryogenic or geomorphological processes. The bone sampled from Yellowstone National Park was collected under permit YELL-SCI-5486. Bone collecting in the Arctic National Wildlife Refuge was done in collaboration with, and by staff from, the Arctic National Wildlife Refuge, US Fish and Wildlife Service.

To calculate the duration each bone persisted on the landscape prior to collection, we first calibrated each radiocarbon date to "years before present" using the 'rcarbon'[3] (version 1.4.2) package in R (version 4.1.1) and the IntCal20 calibration curve[4]. Because the "present" in "years before present" is set to 1950, calibrated ages will underestimate the true weathering duration for a bone collected after 1950 (all bones used in this study). Therefore, to each calibrated age, we added the number of years between 1950 and the date of bone collection. When the date of collection was not available, we used the year that fieldwork was conducted, the publication year, or the year of manuscript submission (Supplementary Data 1).

b. Estimating temperatures at bone, fossil, and eDNA localities.

For each bone, fossil, and eDNA locality, we estimated the mean annual temperature using the 2.5 minute BioClim1 raster[5] and the raster package (version 3.0-7)[6] in R. For each bone, fossil, or eDNA location, we used QGIS (version 3.4)[7] to project it onto an equal area projection prior to calculating a 1 km buffer around each location. We then calculated the median expected temperate (raster value) within that 1 km region. For one eDNA locality along the coast of Northeast Siberia (Yana Bay), we used a 5km buffer to capture enough raster values. When exact coordinates were not available, we used regional estimates that best-matched descriptions found in the publications (Supplementary Data 2). For Wrangel Island, we calculated the median temperature across the entire island.

c. Modelling mammoth extinction using fossil and eDNA time series.

To estimate the year of mammoth extinction, we required time series data for both fossils and eDNA. For eDNA, we used the data available in Wang et al.[1]. To generate a fossil timeseries for

North America, we collated data from a variety of sources[8–10] (Supplementary Data 3). For the Siberian time series, we used a recent compilation of dated mammoth fossils[11]. To estimate extinction, we used the dodo method of Roberts and Solow[12], as executed in the OLE function in the R package 'sExtinct'[13]. This model is particularly useful for our application because it can accommodate multiple "sightings" of a species at a single interval, which is not unusual in the eDNA time series. All models and figures were generated in R, version 4.0.3[14].

2. Supplementary References

1. Wang, Y. *et al.* Late Quaternary dynamics of Arctic biota from ancient environmental genomics. *Nature* **600**, 86–92 (2021).
2. Fernandez-Jalvo, Y. & Andrews, P. *Atlas of taphonomic identifications*. (Springer, 2016).
3. Crema, E. R. & Bevan, A. Inference from large sets of radiocarbon dates: Software and methods. *Radiocarbon* **63**, 23–39 (2021).
4. Reimer, P. J. *et al.* The IntCal20 Northern Hemisphere radiocarbon age calibration curve (0–55 cal kBP). *Radiocarbon* **62**, 725–757 (2020).
5. Fick, S. E. & Hijmans, R. J. WorldClim 2: new 1-km spatial resolution climate surfaces for global land areas. *Int. J. Climatol* **37**, 4302–4315 (2017).
6. Hijmans, R. J. *raster: Geographic Data Analysis and Modeling*. (2019).
7. QGIS Development Team. *QGIS Geographic Information System*. (Open Source Geospatial Foundation, 2009).
8. MacDonald, G. M. *et al.* Pattern of extinction of the woolly mammoth in Beringia. *Nat. Commun.* **3**, 893 (2012).

9. Mann, D. H., Groves, P., Kunz, M. L., Reanier, R. E. & Gaglioti, B. V. Ice-age megafauna in Arctic Alaska: extinction, invasion, survival. *Quat. Sci. Rev.* **70**, 91–108 (2013).

10. Monteath, A. J., Gaglioti, B. V., Edwards, M. E. & Froese, D. Late Pleistocene shrub expansion preceded megafauna turnover and extinctions in eastern Beringia. *Proc. Natl. Acad. Sci. USA.* **118**, e2107977118 (2021).

11. Dehasque, M. *et al.* Combining Bayesian age models and genetics to investigate population dynamics and extinction of the last mammoths in northern Siberia. *Quat. Sci. Rev.* **259**, 106913 (2021).

12. Roberts, D. L. & Solow, A. R. When did the dodo become extinct? *Nature* **426**, 245–245 (2003).

13. Clements, C. F. *et al.* Experimentally testing the accuracy of an extinction estimator: Solow's optimal linear estimation model. *J. Anim. Ecol.* **82**, 345–354 (2013).

14. R Core Team. R: A language and environment for statistical computing. *R Foundation for Statistical Computing, Vienna, Austria* (2018).
