# Supplementary material for: When did mammoths go extinct?
Source: Nature. 2022 Nov 30;612(7938):E1–3. doi: 10.1038/s41586-022-05416-3 (PMC9712083; doi:10.1038/s41586-022-05416-3)
Supplement: Supplementary file 2 — Reporting Summary [file 41586_2022_5416_MOESM2_ESM.pdf]

nature portfolio

Corresponding author(s): Joshua Miller

Last updated by author(s): Mar 29, 2022

Reporting Summary

Nature Portfolio wishes to improve the reproducibility of the work that we publish. This form provides structure for consistency and transparency in reporting. For further information on Nature Portfolio policies, see our Editorial Policies and the Editorial Policy Checklist.

Statistics

For all statistical analyses, confirm that the following items are present in the figure legend, table legend, main text, or Methods section.

n/a	Confirmed	
☐	☒	The exact sample size (n) for each experimental group/condition, given as a discrete number and unit of measurement
☐	☒	A statement on whether measurements were taken from distinct samples or whether the same sample was measured repeatedly
☐	☒	The statistical test(s) used AND whether they are one- or two-sided<br>*Only common tests should be described solely by name; describe more complex techniques in the Methods section.*
☒	☐	A description of all covariates tested
☒	☐	A description of any assumptions or corrections, such as tests of normality and adjustment for multiple comparisons
☐	☒	A full description of the statistical parameters including central tendency (e.g. means) or other basic estimates (e.g. regression coefficient) AND variation (e.g. standard deviation) or associated estimates of uncertainty (e.g. confidence intervals)
☒	☐	For null hypothesis testing, the test statistic (e.g. F, t, r) with confidence intervals, effect sizes, degrees of freedom and P value noted<br>*Give P values as exact values whenever suitable.*
☒	☐	For Bayesian analysis, information on the choice of priors and Markov chain Monte Carlo settings
☒	☐	For hierarchical and complex designs, identification of the appropriate level for tests and full reporting of outcomes
☒	☐	Estimates of effect sizes (e.g. Cohen's d, Pearson's r), indicating how they were calculated

Our web collection on statistics for biologists contains articles on many of the points above.

Software and code

Policy information about availability of computer code

Data collection	We estimated the mean annual temperature of different locations around the world using the 2.5 minute BioClim1 raster (WorldClim2) and QGIS (version 3.4). Values were extracted using the 'raster' (version 3.0-7) package in R (version 4.0.3).
Data analysis	Data were analyzed using the open-source software R (version 4.0.3). Radiocarbon dates were calibrated using the 'rcarbon' (version 1.4.2) package in R. To estimate the timing of extinction, we used the OLE function in the R package 'sExtinct' (version 1.1).

For manuscripts utilizing custom algorithms or software that are central to the research but not yet described in published literature, software must be made available to editors and reviewers. We strongly encourage code deposition in a community repository (e.g. GitHub). See the Nature Portfolio guidelines for submitting code & software for further information.

Data

Policy information about availability of data

All manuscripts must include a data availability statement. This statement should provide the following information, where applicable:

- Accession codes, unique identifiers, or web links for publicly available datasets
- A description of any restrictions on data availability
- For clinical datasets or third party data, please ensure that the statement adheres to our policy

All data generated or analysed in this study are included from this published article (and its supplementary information files) or from Wang et al. 2021 (https://www.nature.com/articles/s41586-021-04016-x).

Field-specific reporting

Please select the one below that is the best fit for your research. If you are not sure, read the appropriate sections before making your selection.

☐ Life sciences ☐ Behavioural & social sciences ☒ Ecological, evolutionary & environmental sciences

For a reference copy of the document with all sections, see nature.com/documents/nr-reporting-summary-flat.pdf

Ecological, evolutionary & environmental sciences study design

All studies must disclose on these points even when the disclosure is negative.

Study description

This study uses radiocarbon ages of bones sitting on landscapes around the world to estimate how mean annual temperature impacts the duration that bones can persist (unburied) in different environmental settings. We compare the resulting expectation of bone persistence duration to evaluate whether middle Holocene sediments containing mammoth DNA may have come from long-dead individuals as their remains decompose (as opposed to DNA contributed by late-surviving mammoths populations).

Research sample

The most severely weathered bones known from Yellowstone National Park and the Arctic National Wildlife Refuge, Alaska are included in this analysis, along with all literature records appropriate for estimating the persistent of bones on different landscapes around the world. Additionally, we include all previously dated and published mammoth bones from Siberia and North America (which come from previously published compilations).

Sampling strategy

Data presented here represent an exhaustive survey for all subjects. This includes known data on bone persistence around the world, all known mammoth bones from Siberia and North America, and all mammoth-bearing eDNA records published from Wang et al. 2021.

Data collection

Data collection on bones from Yellowstone National Park and the Arctic National Wildlife Refuge were collected by Joshua Miller using a standardized data collection scheme. Data on geographic location and taphonomic setting were taken in the field using a GPS and a field notebook. Additional observation on taphonomic state were evaluated in the lab through visual analysis. Miller collected all data related to bone persistence.

Timing and spatial scale

Most of the data used in these analyses come from efforts across many decades (from which all appropriate data are used). The eDNA data come directly from Wang et al. 2021 (all mammoth-bearing eDNA sediments from Siberia and North America are used; https://www.nature.com/articles/s41586-021-04016-x). The bone persistence data similarly come from all available data (which includes samples collected across 63 years).

Data exclusions

No data were excluded from this study.

Reproducibility

Lab experimentation was not part of this work. However, by incorporating data from decades of work, the reproducibility of different portions of our results (e.g., the time period after which mammoth fossils are no longer recovered in Northeast Siberian, Northwest and Central Siberia, and northern North America) is highlighted. Further, all methods are divulged to encourage additional, directly comparable work.

Randomization

Randomization was not relevant for our study. This study specifically evaluates differences in the distribution of fossils and eDNA samples within three specific geographic regions.

Blinding

Blinding was not relevant for our study. This work is largely a reevaluation of published data.

Did the study involve field work? ☒ Yes ☐ No

Field work, collection and transport

Field conditions

Fieldwork (Yellowstone National Park, Arctic National Wildlife Refuge) was conducted on fair-weather days (e.g., not activity raining).

Location

For Yellowstone National Park, bones were evaluated across the Northern Range (approximately: 44.95 latitude, -110.50 longitude). For the Arctic National Wildlife Refuge, bones were evaluated across the Coastal Plain (approximately: 69.63 latitude, -141.43 longitude). Mammoth bones were evaluated across northern North America, and Siberia.

Access & import/export

All new collections highlighted in this work were done in accordance with all local, state, and federal laws. Collection from Yellowstone National Park was done under permit YELL-2007-SCI-5486, approved by Tom Olliff (Chief, Yellowstone Center for Resources) in 2007. Collections from the Arctic National Wildlife Refuge were approved in 2018 by Steve Berendzen (Manager, Arctic National Wildlife Refuge).

Disturbance

No discernible site disturbance was caused by this study. All study sites were approached on foot and all efforts were done to minimize impact during the work.

Reporting for specific materials, systems and methods

We require information from authors about some types of materials, experimental systems and methods used in many studies. Here, indicate whether each material, system or method listed is relevant to your study. If you are not sure if a list item applies to your research, read the appropriate section before selecting a response.

Materials & experimental systems

n/a	Involved in the study
☒	☐ Antibodies
☒	☐ Eukaryotic cell lines
☐	☒ Palaeontology and archaeology
☒	☐ Animals and other organisms
☒	☐ Human research participants
☒	☐ Clinical data
☒	☐ Dual use research of concern

Methods

n/a	Involved in the study
☒	☐ ChIP-seq
☒	☐ Flow cytometry
☒	☐ MRI-based neuroimaging

Palaeontology and Archaeology

Specimen provenance

All new collections highlighted in this work were done in accordance with all local, state, and federal laws. Collection from Yellowstone National Park was done under permit YELL-2007-SCI-5486, approved by Tom Olliff (Chief, Yellowstone Center for Resources) in 2007. Collections from the Arctic National Wildlife Refuge were approved in 2018 by Steve Berendzen (Manager, Arctic National Wildlife Refuge).

Specimen deposition

All specimens are curated at public repositories. Newly published bone specimens highlighted here are available at the Department of Geology's collections, University of Cincinnati, Cincinnati, OH, USA.

Dating methods

AMS radiocarbon dates were generated from collagen extracted using standard acid/base pretreatments. Dates were acquired from the Center for Accelerator Mass Spectrometry (Lawrence Livermore) and University of California Irvine Keck-CCAMS facility. Quality assurance was monitored using internal lab standards and radiocarbon blanks. Radiocarbon dates were calibrated using the ‘rcarbon’ (version 1.4.2) package in R and the IntCal20 calibration curve.

☒ Tick this box to confirm that the raw and calibrated dates are available in the paper or in Supplementary Information.

Ethics oversight

Because all specimens were dead at the time of collection, no ethical oversight or guidance was required for this work.

Note that full information on the approval of the study protocol must also be provided in the manuscript.
